# Aspiration of thrombus for intermediate-risk subacute pulmonary embolism

**DOI:** 10.1186/s13019-024-02648-4

**Published:** 2024-04-08

**Authors:** Jiahao He, Chunli Liu

**Affiliations:** 1 National Center for Respiratory Medicine, Department of Respiratory and Critical Care Medicine, Guangzhou Institute of Respiratory Health, The First Affiliated Hospital of Guangzhou Medical University, Guangzhou, Guangdong China; 2 Department of Respiratory and Critical Care Medicine, Yangjiang Hospital Affiliated of Guangdong Medical University, YangJiang, Guangdong China

**Keywords:** Thrombosis, Pulmonary hypertension, Thrombectomy

## Abstract

Pulmonary embolism is the most common cardiovascular disease after myocardial infarction and stroke. Konstantinides (Eur Heart J 41(4):543–603, 2020) Current guidelines categorize patients with PE as being at low, intermediate, and high risk of early death, with the intermediate-risk group experiencing the greatest uncertainty regarding treatment recommendations. Rapid reduction of the thrombus load by thrombolysis significantly reduces symptoms and decreases mortality, but is accompanied by a high risk of bleeding. Meyer (N Engl J Med 370(15):1402-11, 2014) Mechanical thrombectomy (CDTE) have been proven safe and efficient, yet current ESC guidelines suggest the utilization of catheter interventions only for hypotensive patients with high bleeding risk, failed systemic thrombolysis, and cardiogenic shock or if a patient does not respond to conservative therapy Konstantinides (Eur Heart J 41(4):543–603, 2020). Here, we report a case of an intermediate-risk patient with pulmonary embolism who underwent thrombus aspiration and showed significant improvement in symptoms after treatment.

## Introduction

Treatment in the population with intermediate-risk pulmonary embolism is unclear, and the prevailing view is that catheter intervention should be used only in hypotensive patients with a high risk of bleeding, failure of systemic thrombolysis, cardiogenic shock, or in patients with high-risk pulmonary embolism who are refractory to conservative treatment, and it is unclear whether catheter intervention should be used in the subacute stage of pulmonary embolism. We report a case of intermediate-risk pulmonary embolism in the subacute stage that underwent thrombus aspiration and showed significant improvement in symptoms after treatment.

## Case presentation

**Fig. 1:**
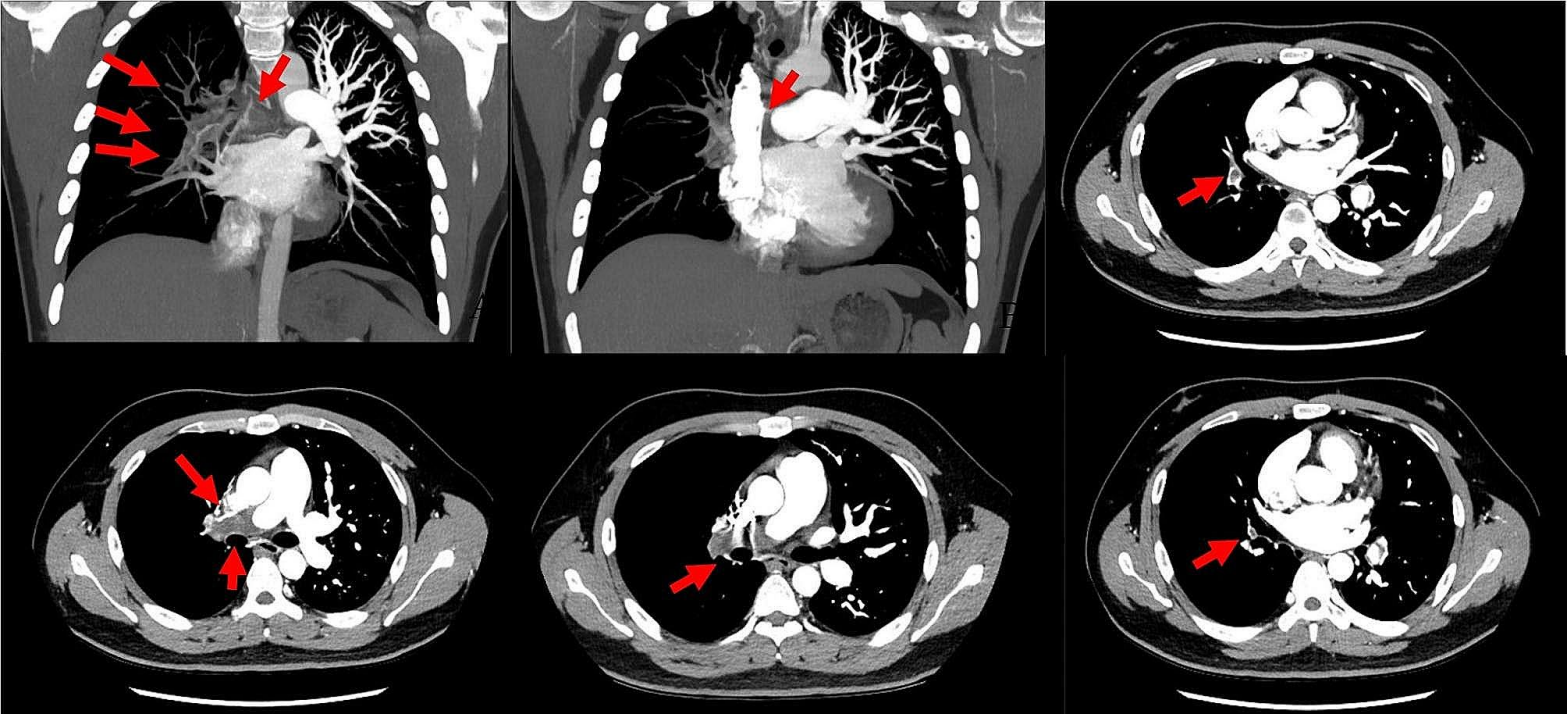
Computed tomographic pulmonary angiography. (**A** to **F**) Multiple patchy and lamellar filling defects in the right main pulmonary artery, right upper middle and lower pulmonary artery and its branches

A 25-year-old male presented with shortness of breath after activity for a month and a half, because of a history of vasculitis, he had been treated with oral hormones for a long time, and for which he had undergone femoral artery prosthetic bypass and right femoral artery endarterectomy 4 years ago. Computed tomographic pulmonary angiography (CTPA) demonstrated multiple emboli in the right main pulmonary artery and the right branch pulmonary artery as well as the left inferior pulmonary artery, which was then diagnosed as intermediate risk for pulmonary embolism (abnormally elevated troponin and NT-proBNP). CTPA was repeated 50 days after receiving adequate low-molecular heparin anticoagulation therapy and showed no significant reduction in pulmonary embolism (Fig. 1). Physical examination, transthoracic echocardiography and lower-limb compression ultrasonography were found no abnormalities. Laboratory tests, including D-dimer, NT-proBNP, markers of myocardial injury, tumor markers of lung cancer, and autoantibody profile were not found to be abnormal. After evaluation by the multidisciplinary pulmonary embolism team, treatment with mechanical thrombectomy was considered due to poor anticoagulation, high surgical risk, and non-exclusion of thromboembolic pulmonary hypertension. Intraoperative pulmonary arteriogram showed complete occlusion of the right main pulmonary artery and incomplete occlusion of the left lower pulmonary artery(Fig. ;2), with a main pulmonary artery pressure of 60/15(32) mmHg. Mechanical thrombectomy was subsequently used to perform thrombus removal in the main pulmonary artery and the right lower pulmonary artery(Fig. ;3), catheter-directed thrombolysis was performed on the distal A1 segment of the right upper pulmonary artery, with a postoperative pulmonary artery pressure of 43/13(25) mmHg. CTPA showed a significant reduction in thrombus 3 days after surgery (Fig. 4), and standard dose rivaroxaban anticoagulation was continued postoperatively. Shortness of breath disappeared at 1 month follow-up, and CTPA demonstrated a reduction in thrombus. (Fig. 5)No adverse events such as bleeding occurred during the treatment.

**Fig. 2:**
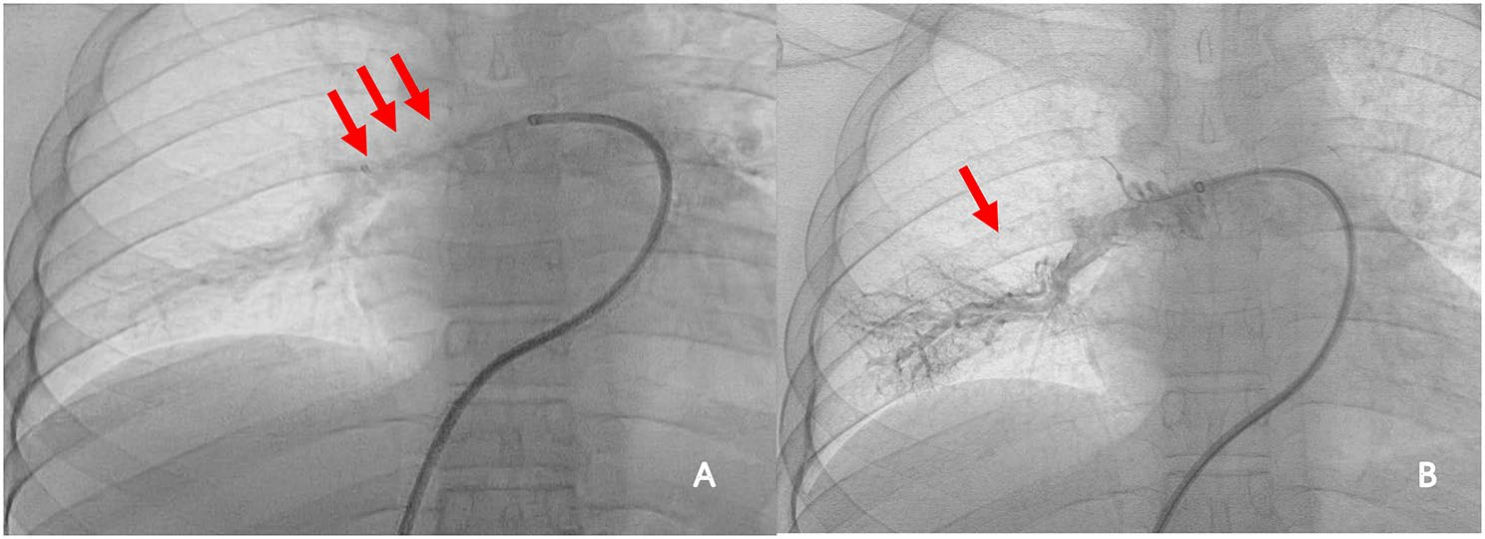
Intraoperative pulmonary arteriogram (**A**) Complete occlusion of the right main pulmonary artery. (**B**) Incomplete occlusion of the left inferior pulmonary artery

**Fig. 3:**
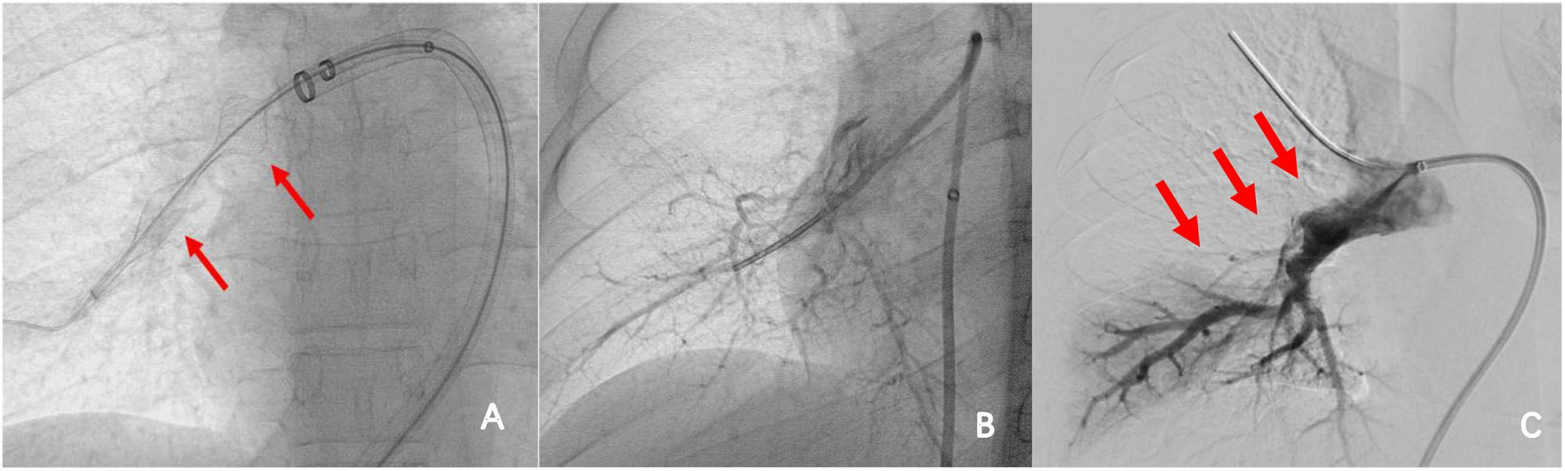
Intraoperative pulmonary arteriogram (**A**) Removal of right lower pulmonary thrombus by mechanical thrombectomy. (**B, C**) Right main pulmonary artery and right inferior pulmonary artery recanalization

**Fig. 4:**
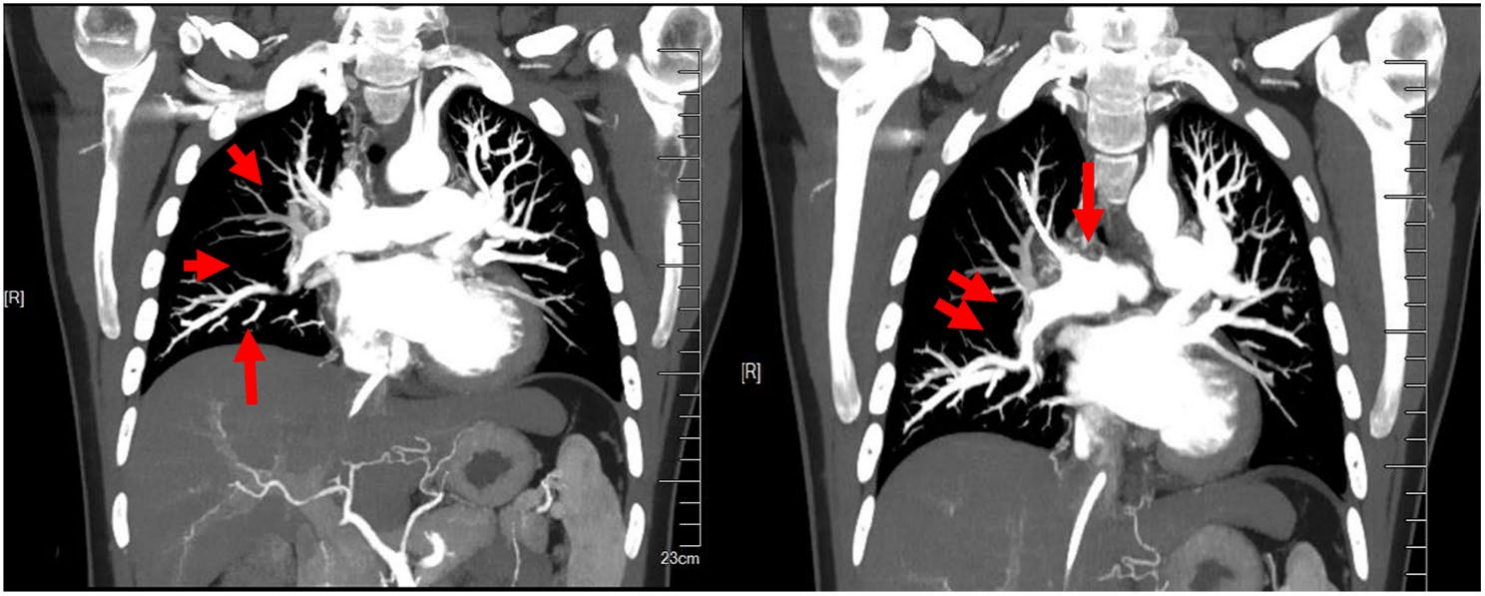
Right main pulmonary artery and right inferior pulmonary artery occlusion opened more than before after thrombus removal, main pulmonary artery stenosis and left inferior pulmonary artery stenosis

**Fig. 5:**
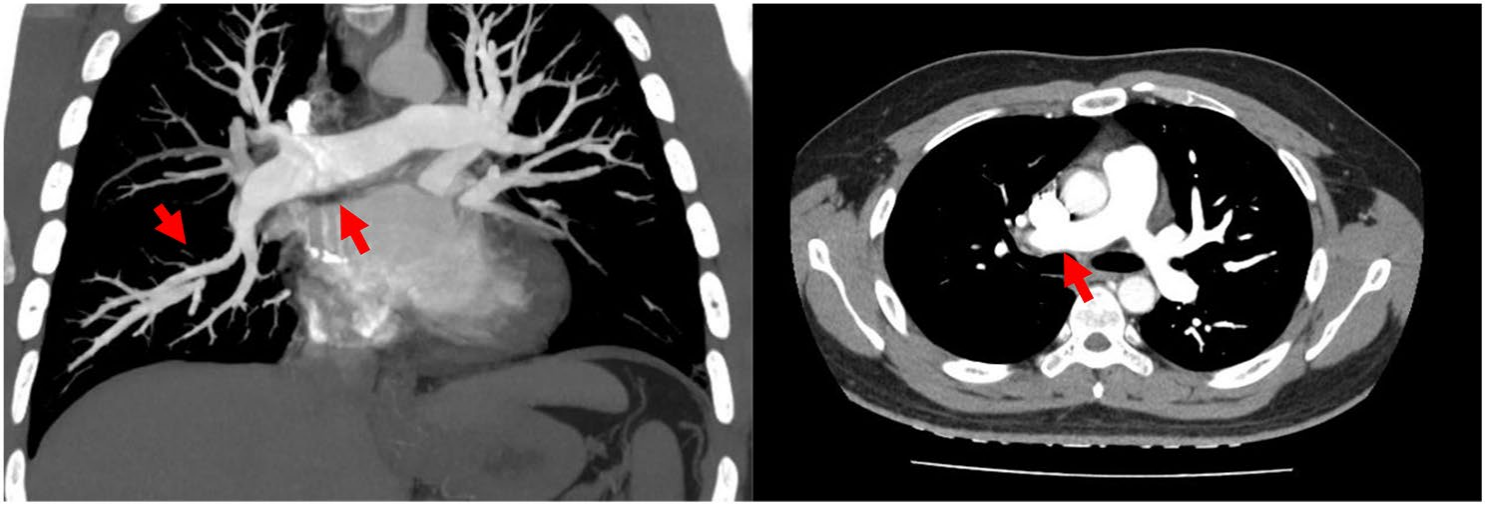
Follow-up CTPA after 1 month: reduction in right pulmonary artery thrombus

## Discussion

The occurrence of pulmonary embolism in this patient was associated with vasculitis, with no significant reduction of thrombus after 50 days of standard treatment, and even suspected pulmonary hypertension, which was subsequently confirmed in a right heart catheter. Catheter-directed thrombectomy is an effective therapeutic option for patients with PE who do not improve hemodynamically and clinically with anticoagulation or who are at risk for secondary deterioration. Mechanical thrombectomy has been previously described as safe and effective for the treatment of acute pulmonary embolism who with high risk of early death [3–5]. In our practice, mechanical thrombectomy in an intermediate-risk patient resulted in a satisfactory outcome and no adverse events. It’s worth noting that the patient in our case was a subacute pulmonary embolism in whom anticoagulation was not effective. To our knowledge, this is the first report that use mechanical thrombectomy for the treatment of subacute pulmonary embolism. Accordingly, we concluded that mechanical thrombectomy has shown promise in reducing the incidence of chronic thromboembolic pulmonary hypertension in patients with pulmonary embolism who have had poor results with anticoagulation therapy.

## Conclusions

In patients in whom anticoagulation is ineffective in the acute phase of pulmonary embolism, the development of chronic thromboembolic pulmonary hypertension can be avoided by using interventional therapy in the subacute phase.

## Data Availability

Not applicable, case report.
